# Dogs' ability to detect an inflammatory immune response in cattle via olfaction

**DOI:** 10.3389/fvets.2024.1393289

**Published:** 2024-04-09

**Authors:** Aiden E. Juge, Nathaniel J. Hall, John T. Richeson, Reinaldo F. Cooke, Courtney L. Daigle

**Affiliations:** ^1^Department of Animal Science, Texas A&M University, College Station, TX, United States; ^2^Department of Animal Science, Texas Tech University, Lubbock, TX, United States; ^3^Department of Agricultural Sciences, West Texas A&M University, Canyon, TX, United States

**Keywords:** canine, working dog, olfaction, disease detection, bovine, cattle, animal health

## Abstract

**Introduction:**

Canine olfaction is a potential means for detection of respiratory disease in beef cattle. In a prior study, two dogs were trained to discriminate between nasal swabs from healthy cattle and cattle that developed Bovine Respiratory Disease. Dogs had some ability to identify samples from BRD-affected cattle, but results were ambiguous. The purpose of this study was to evaluate more dogs using better-controlled training and testing procedures.

**Methods:**

Nasal and saliva swabs were collected from 96 cattle before and after administering a vaccine to induce an inflammatory immune response. Samples were stored at −80°C for up to 11 months before use, and samples from animals with an elevated body temperature at baseline were omitted. An automated olfactometer apparatus was constructed to improve blinding procedures and reduce opportunities for odor contamination. Four dogs were trained to distinguish between swabs from healthy and sickness-model cattle, including the two dogs from the previous study (“Runnels” and “Cheaps”) and two inexperienced dogs (“Molokai” and “Amy”). During a seven-month training period, dogs were exposed to samples from 28 animals. Dogs were tested on 59 sets of unfamiliar samples.

**Results:**

Performance varied among dogs (χ^2^ = 10.48, *p* = 0.02). Molokai's performance was above chance (0.73 ± 0.06, *p* = 0.0006), while Amy (0.44 ± 0.06, *p* = 0.43), Cheaps (0.53 ± 0.07, *p* = 0.79), and Runnels (0.56 ± 0.06, *p* = 0.43) did not respond correctly at a rate different from chance. Accuracy did not differ between nasal swabs (0.63 ± 0.08) and saliva swabs (0.53 ± 0.08, χ^2^ = 0.81, *p* = 0.37).

**Discussion:**

The results of this study indicate that canine olfaction may be an effective means of detecting illness in beef cattle. However, individual dogs' aptitude for this detection task varies.

## 1 Introduction

Detection of bovine respiratory disease (BRD) is a priority in beef cattle production. Treatment of BRD incurs a cost of $23.60 per animal and ~16.2% of cattle produced in large feedlots experience reduced welfare as a result (1). Currently, detection of BRD in feedlot cattle typically relies on the identification of signs of illness that can be visually detected by pen riders from a distance or while riding a horse (2). Illness signs indicative of BRD include behavioral depression, nasal and ocular discharge, and coughing (3, 4) and have been incorporated into clinical illness scoring systems that were designed to take an objective and standardized approach to detecting sick cattle. However, studies of clinical illness scoring have varied in what indicators were included and whether a formalized scoring system with predetermined decision points was used. In addition to lack of consistency, the overall sensitivity of clinical illness scoring for detection of BRD has been estimated at 0.27, indicating that the majority of sick animals are not identified for treatment (3). Because cattle are prey animals, it is adaptive for them to minimize external signs of illness, and therefore sick cattle may easily be missed by human observers (5). Thus, there is a need to develop alternative strategies for detecting BRD.

Dogs can accurately detect numerous bacterial, viral, and neoplastic diseases via olfaction, using substances including breath, mucus, blood, and tissues samples (6, 7). In cattle, dogs are able to detect bovine viral diarrhea virus and staphylococcus aureus infection (8, 9). The basis for olfactory detection of disease is that there are differences in the pattern of volatile organic compounds (VOCs) produced by sick compared to healthy animals. In a prior study, an attempt was made to train two dogs to detect cattle at risk of developing bovine respiratory disease (10). Dogs were trained using positive reinforcement to produce a nose hold alert on a station containing a nasal swab from a bull or steer that was subsequently treated multiple times for BRD or died, while alerts on stations containing a sample from an animal that was not subsequently treated for BRD were not reinforced. In a double-blind test, the dogs were presented with sets of three unfamiliar samples, two negative and one positive. Accuracy was greater than chance for one dog, at 0.45, but no better than chance for the other, at 0.39. Samples were collected as cattle entered the feedlot, and BRD status was determined based on whether the animal was treated for BRD within 20 days of arrival. Consequentially, samples designated positive may have been taken prior to initiation of an inflammatory response, and samples designated negative could include asymptomatic BRD-affected cattle, resulting in ineffective training (10). Lack of consistency in sample handling have been shown to affect disease detection learning in canines (11). Therefore, one objective of the present study was to evaluate canines' performance in detecting a known inflammatory response of beef cattle in a well-controlled context.

Vaccination with a respiratory vaccine containing a strong adjuvant has been validated as a model of the inflammatory response induced by BRD (12). The acute-phase protein haptoglobin was elevated in cattle affected by BRD (13–17) and reached similar levels 24–96 h after vaccination (12). Cortisol was also associated with poor health outcomes in cattle (17, 18), and peaked 2–8 h after vaccination (12). Using a vaccine-challenge model rather than experimental *Mannheimia haemolytica* infection minimizes adverse impact to the welfare of the experimental cattle by minimizing the duration of illness, risk of mortality, and need for invasive monitoring procedures (12). Therefore, using vaccination to induce an inflammatory response facilitates collecting a large number of samples with minimal impact to cattle welfare.

Exposure to multiple samples is key in category-based discrimination learning (19), and sample size is also an important consideration to ensure adequate statistical power for detection test results. Previous canine disease detection studies have used a median of 27 positive and 46 negative samples in training, and 20 positive and 53 negative samples in testing (6). Using samples from the same cattle pre-and post-vaccination will both maximize sample size and reduce extraneous variables within sample sets that could impede dogs' learning about VOCs representative of an inflammatory response. This is analogous to the approach taken by prior studies that have used samples of bacterial cultures rather than patient secretion to demonstrate dogs' ability to detect a given disease in a controlled context (9, 20, 21).

Sample type is also associated with differences in detection success; however, the use of nasal mucus or saliva samples for canine medical detection is relatively rare (6). Another factor that may have impaired dogs' success in the previous pilot study of canines' ability to detect BRD was potential sample contamination with dirt or other substances (e.g., pollen, seasonal differences in feedstuffs) present in cattle's noses (10). To evaluate the effects of sample type on BRD detection success, this study will include both nasal swabs and saliva samples as detection targets.

Several studies of dogs' ability to detect disease have reported individual differences in performance between dogs (8, 22–27). Due to the time investment required for training, it is also common practice to re-use detection dogs across multiple studies (28–34). However, there is a lack of knowledge about how changing training procedures and sample sources could affect performance in a subsequent study. To evaluate whether improved training and sample collection procedures could facilitate illness detection in previously unsuccessful dogs, this study included the two dogs with prior experience and two inexperienced dogs. To evaluate the effect of sample type on detection ability, both nasal and saliva swabs were collected.

## 2 Methods

### 2.1 Study animals

Four dogs were included in this study, all scent-hound type mixed-breeds. Furher information about dogs' ancestry was unknown. Dog-related procedures were approved under Texas A&M University Institutional Animal Care and Use Committee Protocol #2020-0299. All dogs were sourced from the Texas Department of Criminal Justice, where they were initially trained for scent tracking but had been deemed unsuitable for the program. Dogs were singly housed at the Texas A&M University (TAMU) Comparative Medicine and Pathology facility, in kennels measuring 2.5 m × 2.2 m × 2.5 m. Kennels were illuminated by fluorescent lights on a 12 h light/12 h dark schedule and were cleaned twice daily on weekdays and once daily on weekends and holidays. Dogs were fed 5L18 kibble (LabDiet, St. Louis, MO, USA) twice daily, with quantities adjusted monthly as needed to maintain each dog at a healthy weight.

Two dogs, Runnels and Cheaps, were included in a prior study of dogs' ability to detect BRD (10). At the time of the current study, Runnels was 8 years old, and had resided at the kennel facility for 7 years, and Cheaps was 6 years old, and had resided at the kennel facility for 5 years. Both dogs had previously been part of the teaching colony at the TAMU College of Veterinary Medicine and were trained in basic cues via clicker training and positive reinforcement. Two additional dogs were added for this study. Initially, two dogs were selected from a group of four available dogs from the Texas Department of Criminal Justice and were moved to the TAMU Comparative Medicine and Pathology facility 2 months prior to the start of detection training. Amy, a spayed female, was 6 years old at the time of this study, and Festus, a neutered male, was 4 years old at the time of this study. Both dogs underwent a 2-week quarantine and were subsequently trained in basic obedience cues and leash walking using positive reinforcement. However, Festus was eliminated from the project due to fear of humans and lack of interest in training. Molokai, a 6-year-old neutered male, was subsequently added to the study. He had resided at the kennel facility for 2 years as part of the teaching colony and had prior experience with clicker training and positive reinforcement, but no prior scent detection training.

### 2.2 Sample acquisition and storage

Nasal and saliva swab samples were collected from 96 steers housed at the Texas A&M McGregor Research Station in McGregor, Texas. Cattle procedures were approved under Texas A&M University Agricultural Animal Care and Use Committee Protocol #2022-011A. Four weeks after weaning, cattle were vaccinated with a modified live-virus respiratory vaccine, Bovishield One Shot Gold (Zoetis). This method has been shown to induce a sickness-like inflammatory response (12). Nasal and saliva swab samples were collected immediately prior to vaccination (VAX) and 29–30 h after vaccination (VAX30), at the anticipated haptoglobin peak. Additional swab samples were collected from 20 focal animals, which included one–two animals from each of 16 pens, 5–6 h after vaccination (VAX6), at the anticipated cortisol peak.

To validate the vaccine as a model of illness, temperature was measured for all steers and serum samples were collected from the 20 focal steers at the same time points as swab collection. Serum samples were sent to the Kansas State Veterinary Diagnostic Lab (Manhattan, KS) for haptoglobin assay and to the Texas Veterinary Medical Diagnostic Lab (College Station, TX) for cortisol assay. Temperature, haptoglobin, and cortisol levels increased from the first to the third sample collection time (unpublished data). Changes in temperature and serum haptoglobin confirmed the presence of an inflammatory immune response. Swabs from nine cattle were excluded from the training process due to baseline temperature of >40°C indicating a high likelihood of pre-existing illness.

At each sample collection point, three nasal swabs and three saliva swabs were collected from each animal, resulting in a total of 12 samples collected from each steer, and an additional six samples from each of the 20 focal animals. All saliva and nasal swab samples were collected by the same person, who wore clean nitrile gloves that were changed between animals. For nasal swab samples, a bundle of three 6″ cotton-tipped wooden swabs (VWR, Radnor, PA) were inserted into the steer's left nostril at a depth of 2–3″ and swirled until saturated. For saliva swab samples, an identical bundle of three swabs was inserted into the steer's mouth and rubbed against the tongue until saturated. Swabs were immediately placed into clean 40 ml amber glass vials (QEC, Beaver, WV), the sticks were trimmed, and the vials were sealed and placed in coolers, on ice packs.

Immediately after each sample collection session, which were 3–4 h in duration, samples were frozen at −4°C. One day after all samples were collected, samples were transported on dry ice to College Station, TX, where they were transferred to an ultralow freezer (VWR, Radnor, PA) for storage at −80°C. Samples were stored for 4–11 months before use. Samples were thawed the morning of the first day they were to be used and placed into separate clean 20 ml vials for use with the olfactometers. Samples remained in the olfactometer at room temperature, 16°C−27°C, for the duration of time in which they were used.

### 2.3 Olfactometer

A lineup of three olfactometer devices were used to train dogs and evaluate performance. The olfactometers were constructed following a modified version of the design described by Aviles-Rosa et al. (35). Each olfactometer was a battery-operated standalone unit including an air pump, filter, air flow meters, attachment points for up to six sample vials, and a detection port with an IR beam break sensor. Each olfactometer incorporated an Arduino Nano BLE 33 microcontroller (Arduino, Somverville, MA, USA), which interfaced via Bluetooth with a computer running the programs created by Aviles-Rosa et al. (35) to control the olfactometers and record data.

### 2.4 Training room

Dog training and testing was conducted in the same facility as a previous study (10). The training room was 3.66 m × 4.27 m and was maintained between 16 and 27°C. The experimenter stood near the back of the room, which included computer equipment, storage cabinets for supplies, and a sink. The olfactometer boxes were positioned in a line, 1 m apart, against the wall opposite the experimenter. During training, the experimenter encouraged the dogs to search the olfactometers during trials and wait away from the olfactometers between trials.

### 2.5 Cleaning procedures

Olfactometer ports were wiped with methanol between dogs, which was allowed to evaporate for at least 60 s. The training room was swept at least twice a week and mopped weekly and as needed. Between uses, vials and PTFE tubing were washed with unscented dish soap and hot water, dried in an oven at 135°C for 30 min, and then rinsed with methanol. Empty clean vials were left open for 5 min to allow the methanol to evaporate and subsequently placed upside down in a clean storage box. New lids were used for all samples. Study personnel always wore nitrile gloves when handling vials or tubing.

### 2.6 Reinforcement

Throughout the training and testing procedures, dogs' correct responses were reinforced with food rewards. An informal preference test was conducted with each dog prior to the start of training, in which dogs were offered a choice among two types of dry food, canned food, Milk-Bones, soft chicken-flavored treats, and peanut butter placed in a circle on the floor. This exercise was repeated three times for each dog, with the position of each food varied between repetitions. Molokai preferred chicken-flavored treats (Zuke's Mini Naturals, Zuke's, Durango, CO, USA), and all other dogs preferred peanut butter. Dogs' preferred foods were used as food odors and as reinforcers during training.

### 2.7 Dog training

Training methods built on those used in a prior study (10). Each dog underwent training 4–5 days per week, two 20-trial sessions per day, over a 7-month period. The order in which dogs completed training sessions was randomized daily. Dogs were trained on combined nasal and saliva samples, e.g., a nasal and a saliva swab collected from the same animal, at the same time, were placed in a single vial. Odor lines were set to a flow rate of 1 L/min and clean air lines were set to a flow rate of 0.5 L/min. No sample was used for more than a week. Use of an olfactometer limited the samples' exposure to air or other contaminants.

Phase I of training involved familiarizing the dogs with the olfactometer devices and detection task. During Step 1, one olfactometer presented a food odor during each trial and the other two were empty. The required alert time for reinforcement was set to 0.1 s, and dogs were allowed to continue sniffing if an incorrect odor port was selected. In this step of training, the olfactometers were set to “Wait for correct response” due to the short alert time required, so the trial did not end if the dog sniffed an incorrect odor. When the correct odor was selected, dogs were provided with a food reward and the trial ended, initiating a 20 s inter-trial interval. If dogs did not select the correct odor port within 45 s, the trial ended with no reinforcement. Once dogs reached an accuracy rate of 65% within a session (13/20 correct), they advanced to the next step. In Step 2, the required alert time was increased incrementally to 1 s, via gradual addition of randomized variation in alert time, to minimize dog frustration. In step 3, the alert time was incrementally lengthened to 4 s. In Step 4, a 4 s alert was required, and “Wait for correct” was deactivated, so dogs were not allowed to continue searching after an incorrect response.

During Phase II of training, the food odor was replaced by an artificial scent, isoamyl acetate diluted in mineral oil at a concentration of 10^−4^. Steps 1–4 were repeated with the new odor; however, three out of four dogs (Amy, Runnels, and Molokai) never reached the accuracy criterion to progress. Because detection of isoamyl acetate was not directly relevant to the project's goals, the accuracy criteria for this phase were ultimately waived.

In Phase III of training, during each trial, one olfactometer presented odor from a sample collected 30 h post-vaccination, while the other two presented odor from two empty vials. Steps 2, 3, and 4 were repeated at this phase. In Phase IV, the target odor was a sample collected 30 h post-vaccination, with distractor odors including another sample from the same animal at the time of vaccination and an empty vial. During this phase, each trial included one target odor and any combination of the two distractors (e.g., some trials included the pre-vaccination sample and the blank, some had two pre-vaccination samples, and some had two blanks). Training steps 2, 3, and 4 were repeated as in Phase III. Phases III and IV lasted a total of 13 weeks, during which 28 sample sets were used. Due to technical difficulties, during some training sessions, only two olfactometers were available. In Phase III and IV, trials with only two olfactometers did not include an empty vial as a distractor.

### 2.8 Detection test

Samples from 59 steers were reserved for a double-blind detection test. Tests were conducted in the same room as the training sessions. The same olfactometer system was used as was in training, with three samples presented to the dog during each trial. However, the olfactometer software was modified so that each trial included one target odor, one pre-vaccination distractor, and one empty vial. The olfactometer program did not indicate the location of the correct odor to the experimenter, only whether the dog should receive a reward after the trial was complete.

Each dog was tested on all 59 sets of samples. To prevent dog fatigue, sessions occurred on 29 days, over a 6-week period. Dog order was randomized each day to mitigate effects of any sample deterioration. Two sample sets were used during each day of testing, except on day 20, when three sets were used. Each dog was tested on 39 sets of combined nasal and saliva swab samples and completed a 20-trial session with each sample set. For the remaining 20 sample sets, each dog completed 10 trials with saliva swabs only and 10 trials with nasal swabs only.

To control for transfer of learning from the nasal swab task to the saliva swab task or vice versa, two dogs were presented with nasal swabs first and two dogs were presented with saliva swabs first for each sample set. Assignments of dogs to sample types were balanced and pseudorandomized across the 20 sample sets. Additionally, the positions of nasal and saliva swab sample vials in the olfactometer were changed between sample sets. For instance, the first sample set for which nasal and saliva swabs were separated had the saliva swabs placed in valves 1 and 3 of the olfactometers, and the nasal swabs in valves 2 and 4. These positions were reversed for the second set, with nasal swabs in valves 1 and 3 and saliva swabs in valves 2 and 4.

### 2.9 Data analysis

Only dogs' responses on the first trial of each test session was considered in evaluating performance. For all tests, results were considered significant at *p* < 0.05 and tendencies at *p* < 0.10.

Dog performance was reported as the proportion of samples correct and the standard error. Binomial exact tests were performed to determine whether dogs' accuracy differed from chance. Chance accuracy was set at 0.5 as a conservative estimate, because each trial included only one target and one distractor sample, and dogs were not expected to alert on the third, odorless olfactometer at the same rate as the target and distractor samples. Binomial tests were calculated in Microsoft Excel Version 16.42 (Microsoft, Redmond, WA). PROC NPAR1WAY in SAS (SAS Institute Inc, Cary, NC) was used to perform Kruskal-Wallis tests to identify differences in accuracy between dogs, between dogs' first and second training session of the day, between nasal and saliva samples, and between the first and second type of swab presented to the dog. In tests with more than two classes, means were compared via the Dwass-Steel-Critchlow-Fligner (DSCF) method. Numerical results were reported as mean ± standard error.

To evaluate the extent to which dogs' responses to each sample were consistent with each other, responses to each sample set were classified by how many dogs responded correctly. The frequency of each response pattern was compared to the frequency expected by chance via an exact test for goodness of fit. Dogs' collective accuracy was assessed by scoring a correct response by 3 or 4 dogs as “1,” a correct response by two dogs as “0.5,” and a correct response by 0 or 1 dogs as “0.” The mean of these scores was the consensus accuracy. A binomial test was used to determine whether consensus accuracy differed from chance.

To determine whether dogs responded preferentially to particular olfactometers, the dogs' response frequency at each olfactometer was compared to the frequency expected by chance via an exact test for goodness of fit.

## 3 Results

**Figure 1:**
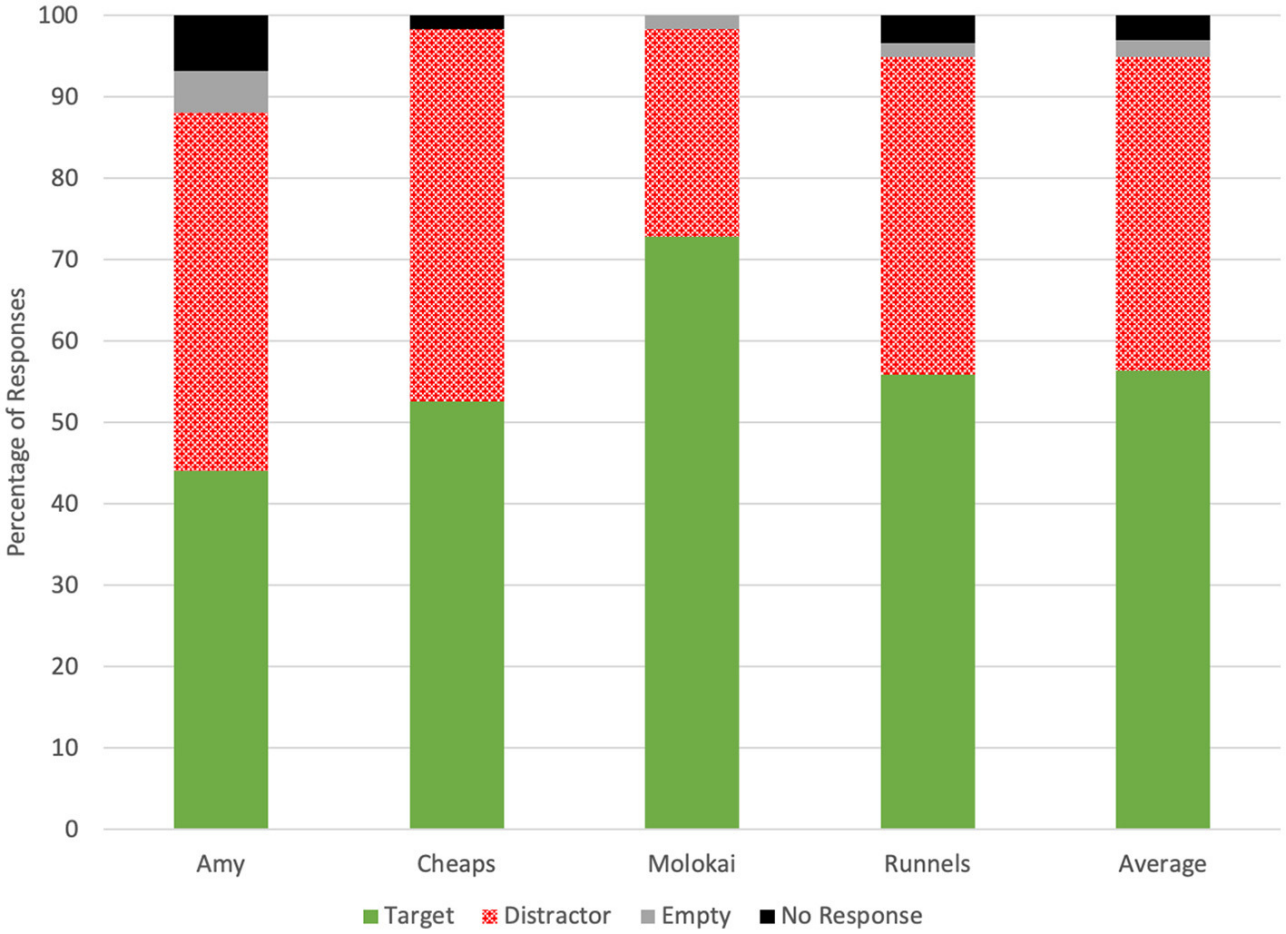
Dogs' frequency of responses to target, distractor, and no-odor samples and the frequency of non-response on the first trial of each test sessi.

**Figure 2:**
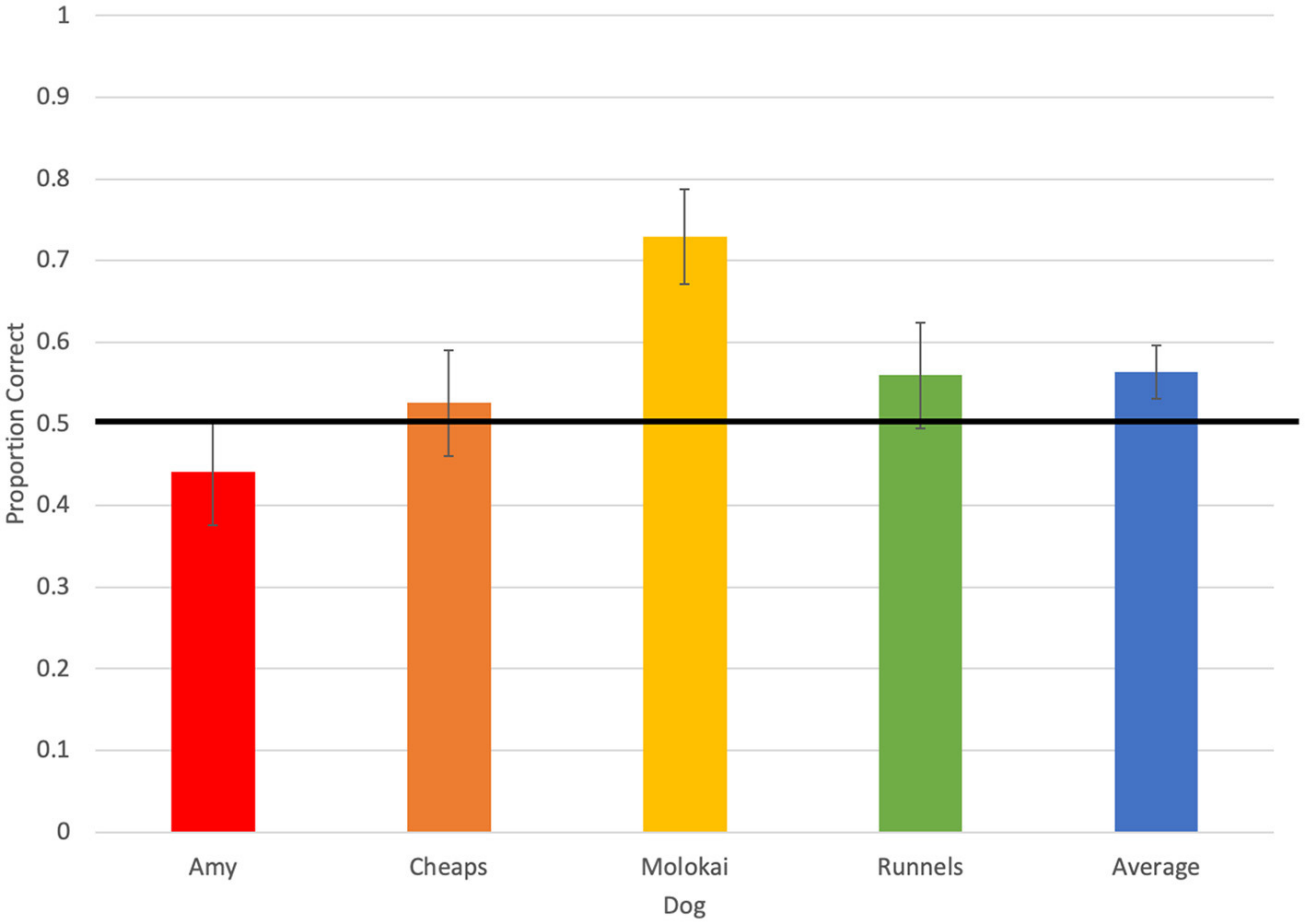
Average accuracy of each of four dogs, and overall average accuracy, on the first trial in each test session. The horizontal line indicates accuracy expected by chance.

Throughout the 236 test trials evaluated for accuracy, dogs selected the olfactometer with no odor five times (2.11%), selected the distractor odor 91 times (38.5%), selected the target odor 133 times (56.4%), and did not respond seven times (2.97%; Figure 1). Average accuracy across all four dogs tended to be greater than chance (0.56 ± 0.03, *p* = 0.06; Figure 2). Individually, however, Molokai's performance was above chance (0.73 ± 0.06, *p* = 0.0006), while Amy (0.44 ± 0.06, *p* = 0.43), Cheaps (0.53 ± 0.07, *p* = 0.79), and Runnels (0.56 ± 0.06, *p* = 0.43) did not respond correctly at a rate different from chance. There were differences in performance between dogs (χ^2^ = 10.48, *p* = 0.02), however, the only significant pairwise difference was between Molokai and Amy (DSCF = 4.47, *p* = 0.009).

Average accuracy in dogs' first session of the day was 0.61 ± 0.05, and accuracy in the second session was 0.52 ± 0.05. Accuracy between session times did not differ (χ^2^ = 2.17, *p* = 0.14). Also, accuracy did not vary between nasal swabs (0.63 ± 0.08) and saliva swabs (0.53 ± 0.08, χ^2^ = 0.81, *p* = 0.37), neither of which differed from chance accuracy (Table 1).

**Table 1:** Dogs' average proportions of correct responses on nasal and saliva swabs on the first trial in each test session.

	**Saliva**	**Nasal**
Amy	0.5	0.3
Cheaps	0.5	0.6
Molokai	0.7	0.8
Runnels	0.4	0.8
Average	0.53	0.63

To evaluate whether dogs transferred learning about one sample type to responding to a different sample type, dogs' performance on the first trial of each half of the “Split Test” sessions was compared. Dogs' mean accuracy on the first sample type they encountered was 0.58 ± 0.06, and accuracy on the second sample type they encountered was 0.61 ± 0.05. There was no difference in performance (χ^2^ = 0.23, *p* = 0.63), indicating that prior exposure to a different type of sample from the same animal did not impact dogs' performance.

To determine whether dogs collectively responded correctly to some samples more than others, responses were evaluated for consistency between dogs. With a cohort of four dogs, there were five possible accuracy outcomes for each sample set (0, 1, 2, 3, or 4 correct). The expected likelihood of each outcome was calculated (0.063, 0.25, 0.375, 0.24, 0.063), and this distribution was compared to the actual frequency of response outcomes (2, 5, 9, 18, 25). Dogs did not agree with each other more often than expected by chance (χ^2^ = 4.57, *p* = 0.33).

The performance of all four dogs as a group was calculated from the number of sample sets for which more than half the dogs responded correctly (scored as 1), half the dogs responded correctly (scored as 0.5), or no dogs responded correctly (scored as 0). The mean score, or consensus accuracy, was 0.60 ± 0.06 and did not differ from chance (*p* = 0.12).

**Figure 3:**
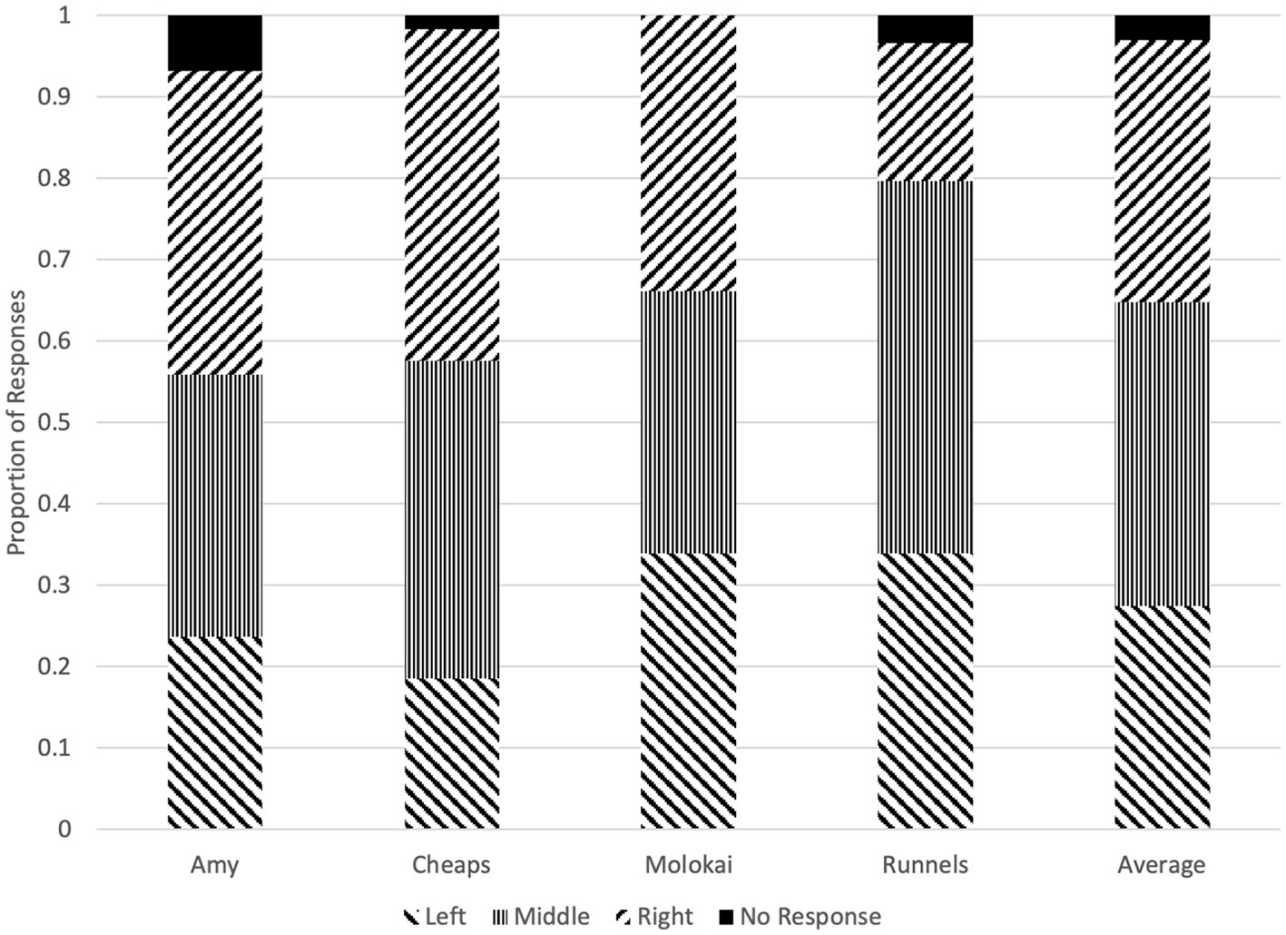
Proportion of responses at each olfactometer made by each dog on the first trial of each test session.

There was no evidence of bias toward a particular olfactometer from Amy (χ^2^ = 1.78, *p* = 0.45), Cheaps (χ^2^ = 5.41, *p* = 0.07), or Molokai (χ^2^ = 0.03, *p* = 1.00). However, Runnels did not respond equally to the three olfactometers (χ^2^ = 7.68, *p* = 0.02), and selected the middle olfactometer in 45.8% of trials and the right-hand olfactometer in 16.9% of trials (Figure 3).

## 4 Discussion

In this study, four dogs were trained to alert on nasal and saliva swab samples from cattle undergoing an inflammatory immune response. In double-blind tests with novel samples, neither the mean accuracy of all four dogs nor the collective accuracy of the four dogs if considered as a panel differed from chance. One dog correctly identified swabs at a rate greater than chance, while the other three did not. While Runnels and Cheaps' numerical accuracy increased from the previous study, this was due to design changes. Previously, dogs were presented with two negative samples and one positive in each trial so chance accuracy was 0.33 (10). In this study, each test trial included one target, one distractor, and one unscented station, which dogs were not expected to select as often as the cattle swabs, if at all (Figure 1). This produced a chance accuracy rate of ~0.5. Notably, the dog that exceeded chance accuracy in the prior study, Cheaps (10), was the only dog to respond well to isoamyl acetate during training. Nonetheless, he did not exceed chance accuracy in distinguishing between pre-vaccination and post-vaccination cattle samples; while Molokai, an inexperienced dog, was successful. Differences in individual dogs' ability to generalize from training samples to test samples in a disease detection paradigm are not unusual. For instance, Grandjean et al. (36) trained 14 dogs for COVID-19 detection, only six of which progressed to testing. Similarly, in a cancer-detection study, two of 10 dogs learned to distinguish between familiar case and control samples, and neither of those successfully identified odors from unfamiliar samples (37). In dogs with prior scent detection experience, three of four dogs failed to generalize a cancer odor across multiple urine samples (24). Therefore, one dog's success at identifying swabs from cattle undergoing a sickness-like immune response indicates that there was an inflammation-related pattern of changes in volatile organic compounds (VOCs) present in bovine nasal and saliva samples, even though not all dogs successfully identified it.

The presence of different concentrations of volatile organic compounds in the breath of healthy cattle and cattle with BRD has been documented in several studies. Potential VOC markers of bovine respiratory disease include increased acetaldehyde and decanal (38) and decreased phenol, benzothiazole, *p-*cresol, and 5-octadecanal (39). However, the presence of specific VOCs can vary depending on the species and strain of pathogen(s) present (40, 41) making VOC-based detection of BRD, which involves multiple pathogens, particularly challenging.

The results of this study cannot be directly compared to evaluations of the accuracy of clinical illness (CI) scoring by humans. The vaccination challenge protocol used in this study simulates animals' immune response during the initial period of illness, which was chosen because developing a screening for detection of BRD prior to the onset of severe symptoms, in order to allow early treatment, is of interest to cattle producers. Studies of CI scoring, however, typically involve evaluation of spontaneously or experimentally infected animals in field settings (3, 42), in which animals may exhibit more severe symptoms of illness. Additionally, in this study, dogs compared samples collected from the same animal at different points in time, rather than samples from multiple animals, and were required to choose between one of two samples, rather than making independent decisions about the health status of each animal.

A second objective of this study was to compare the efficacy of nasal and saliva swabs as detection targets. In a previous study, two of the four dogs had been trained in olfactory detection of BRD using nasal swabs only (10). In this study, in which dogs were trained using combined nasal and saliva swab samples, accuracy of dogs' first response to unfamiliar samples did not differ between nasal and saliva swabs. Numerically, accuracy was higher with nasal swabs than with saliva swabs, and patterns of accuracy by swab type appeared to vary between dogs- Runnels had twice as many correct responses on nasal swabs as saliva swabs, while accuracy on each swab type was similar for Cheaps and Molokai, and Amy responded correctly to more saliva swabs than nasal swabs. However, testing with separate nasal and saliva swabs was performed using relatively few samples, limiting the possibilities for statistical analysis at the level of individual dogs, and limiting the power of the overall comparison of accuracy by swab type. Therefore, it is possible that investigations using a larger sample size could be more informative. Additionally, dogs' performance on the first sample type they encountered from a given animal was similar to performance on second sample type they encountered, indicating that there was no transfer of learning about a specific animal's sickness-related VOC signature across sample types. Nasopharyngeal swabs have previously been used in canine COVID-19 detection studies, with sensitivity of 65%−82.6% and specificity of 89%−96.4% (43, 44). However, nasal and saliva swabs are rarely used in canine olfaction studies (6) and have not previously been compared.

Dogs' agreement with each other did not differ from chance, indicating that there was no pattern of increased or decreased accuracy on particular samples. This corresponds with the findings that three of the four dogs were responding essentially at random and suggests that there was no pattern of olfactory stimuli that made some samples “easier” than others to identify. However, further research is warranted to investigate whether the responses of the one dog that did perform more accurately than chance correspond with other indicators of illness in the animals from which the samples were collected.

Time of day did not affect dogs' accuracy, as dogs performed equally well in their first and second test session of the day. Panting, a result of exercise and high environmental temperature, interferes with dogs' ability to respond correctly during scent detection tasks (45). In this study, dogs were given a period of outdoor exercise prior to each training or test session; however, due to the time of day, outdoor temperature was higher during the exercise period prior to dogs' second test session each day. The lack of performance decrement in the second session indicates that the time when dogs were allowed to rest indoors while the olfactometer was set up for each test session was sufficient to mitigate the effects of outdoor exercise.

Most dogs did not respond disproportionately to any specific olfactometer, indicating that odor contamination or repetitive behavioral patterns resulting in a biased response were not likely to be causes of poor performance.

The results of this study are a promising indicator that dogs have the capacity to identify a sickness-related olfactory signature in cattle. However, only one dog was successful in distinguishing between healthy and sickness-model samples, underscoring the importance of dog selection in training for olfactory detection tasks. There are links between dog personality and performance in a variety of Working careers: trainability, fearlessness, and low reactivity to touch are predictive of success (46). Motor inhibitory control and persistence in searching are linked with accuracy in explosives detection (47). Detection dog performance is also associated with success on cognitive tasks assessing memory, response to human cues, and odor discrimination (48). Therefore, using a battery of behavioral and cognitive tests to select dogs for detection training could increase success rates in future studies. Additionally, the olfactometer devices used in this study collect a variety of data about dogs' patterns of search and response behavior (35). There is evidence that dogs' sniffing durations differ between correct and incorrect responses during a detection test (49). Examining other characteristics of dogs' responses could also be informative, and further analysis of dogs' behavior is planned as part of a future project.

## Data availability statement

The raw data supporting the conclusions of this article will be made available by the authors, without undue reservation.

## Ethics statement

The animal study was approved by Texas A&M University Institutional Animal Care and Use Committee and the Texas A&M University Agricultural Animal Care and Use Committee. The study was conducted in accordance with the local legislation and institutional requirements.

## Author contributions

AJ: Conceptualization, Data curation, Formal analysis, Investigation, Methodology, Writing – original draft. NH: Conceptualization, Methodology, Software, Writing – review & editing. JR: Conceptualization, Writing – review & editing. RC: Methodology, Writing – review & editing. CD: Conceptualization, Funding acquisition, Supervision, Writing – review & editing.

## References

[1] USDA. Feedlot 2011 Part IV: Health and Health Management on US feedlots with a capacity of 1000 or more Head. (2013). Available online at: https://www.aphis.usda.gov/animalhealth/nahms/feedlot/downloads/feedlot2011/Feed11drPartIV.pdf (accessed September 8, 2020).

[2] Richeson JT. Behavior assessment and applications for BRD diagnosis: beef. Anim Health Res Rev. (2020) 21:192–5. 10.1017/S1466252320000249 33682665

[3] Timsit E Dendukuri N Schiller I Buczinski S. Diagnostic accuracy of clinical illness for bovine respiratory disease (BRD) diagnosis in beef cattle placed in feedlots: a systematic literature review and hierarchical Bayesian latent-class meta-analysis. Prev Vet Med. (2016) 135:67–73. 10.1016/j.prevetmed.2016.11.006 27931931

[4] Pillen JL Pinedo PJ Ives SE Covey TL Naikare HK Richeson JT. Alteration of activity variables relative to clinical diagnosis of bovine respiratory disease in newly received feed lot cattle. Bov Pract. (2016) 50:1–8. 10.21423/bovine-vol50no1p1-8

[5] Weary DM Huzzey JM von Keyserlingk MAG. Board-invited review using behavior to predict and identify ill health in animals1. J Anim Sci. (2009) 87:770–7. 10.2527/jas.2008-1297 18952731

[6] Juge AE Foster MF Daigle CL. Canine olfaction as a disease detection technology: a systematic review. Appl Anim Behav Sci. (2022) 253:105664. 10.1016/j.applanim.2022.105664

[7] Edwards TL Browne CM Schoon A Cox C Poling A. Animal olfactory detection of human diseases: guidelines and systematic review. J Vet Behav. (2017) 20:59–73. 10.1016/j.jveb.2017.05.002

[8] Angle TC Passler T Waggoner PL Fischer TD Rogers B Galik PK. Real-time detection of a virus using detection dogs. Front Vet Sci. (2016) 2:79. 10.3389/fvets.2015.00079 26779494 PMC4705269

[9] Fischer-Tenhagen C Theby V Krömker V Heuwieser W. Detecting *Staphylococcus aureus* in milk from dairy cows using sniffer dogs. J Dairy Sci. (2018) 101:4317–24. 10.3168/jds.2017-14100 29501329

[10] Juge AE Hall NJ Richeson JT Daigle CL. Using canine olfaction to detect bovine respiratory disease: a pilot study. Front Vet Sci. (2022) 9:902151. 10.3389/fvets.2022.902151 35847637 PMC9284318

[11] Guest CM Harris R Anjum I Concha AR Rooney NJ A. Lesson in standardization – subtle aspects of the processing of samples can greatly affect dogs' learning. Front Vet Sci. (2020) 7:525. 10.3389/fvets.2020.00525 33015138 PMC7461772

[12] Rodrigues MC Cooke RF Marques RS Cappellozza BI Arispe SA Keisler DH. Effects of vaccination against respiratory pathogens on feed intake, metabolic, and inflammatory responses in beef heifers1. J Anim Sci. (2015) 93:4443–52. 10.2527/jas.2015-9277 26440344

[13] Idoate I Vander Ley B Schultz L Heller M. Acute phase proteins in naturally occurring respiratory disease of feedlot cattle. Vet Immunol Immunopathol. (2015) 163:221–6. 10.1016/j.vetimm.2014.12.006 25599608

[14] Moisá SJ Aly SS Lehenbauer TW Love WJ Rossitto PV Van Eenennaam AL. Association of plasma haptoglobin concentration and other biomarkers with bovine respiratory disease status in pre-weaned dairy calves. J Vet Diagn Invest. (2019) 31:40–6. 10.1177/1040638718807242 30328386 PMC6505765

[15] Joshi V Gupta VK Bhanuprakash AG Mandal RSK Dimri U Ajith Y. Haptoglobin and serum amyloid A as putative biomarker candidates of naturally occurring bovine respiratory disease in dairy calves. Microb Pathog. (2018) 116:33–7. 10.1016/j.micpath.2018.01.001 29330058

[16] Dörtkardeş AB SahiNduran S. Determination of serum amyloid A, haptoglobin and hepcidin levels in calves with endemic viral pneumonia. Ank Üniversitesi Vet Fakültesi Derg. (2020). 10.33988/auvfd.523958

[17] Wisnieski L Amrine DE Renter DG. Predictive modeling of bovine respiratory disease outcomes in feedlot cattle: a narrative review. Livest Sci. (2021) 251:104666. 10.1016/j.livsci.2021.104666

[18] Masmeijer C Deprez P van Leenen K De Cremer L Cox E Devriendt B. Arrival cortisol measurement in veal calves and its association with body weight, protein fractions, animal health and performance. Prev Vet Med. (2021) 187:105251. 10.1016/j.prevetmed.2020.105251 33418516

[19] Moser AY Bizo L Brown WY. Olfactory generalization in detector dogs. Animals. (2019) 9:702. 10.3390/ani9090702 31546835 PMC6769875

[20] Koivusalo M Vermeiren C Yuen J Reeve C Gadbois S Katz K. Canine scent detection as a tool to distinguish meticillin-resistant *Staphylococcus aureus*. J Hosp Infect. (2017) 96:93–5. 10.1016/j.jhin.2017.03.005 28389090

[21] Davies JC Alton E Simbo A Murphy R Seth I Williams K. Training dogs to differentiate *Pseudomonas aeruginosa* from other cystic fibrosis bacterial pathogens: not to be sniffed at? Eur Respir J. (2019) 54:1900970. 10.1183/13993003.00970-2019 31413160

[22] Catala A Grandgeorge M Schaff JL Cousillas H Hausberger M Cattet J. Dogs demonstrate the existence of an epileptic seizure odour in humans. Sci Rep. (2019) 9:4103. 10.1038/s41598-019-40721-4 30923326 PMC6438971

[23] Dehlinger K Tarnowski K House JL Los E Hanavan K Bustamante B. Can trained dogs detect a hypoglycemic scent in patients with type 1 diabetes? Diabetes Care. (2013) 36:e98–9. 10.2337/dc12-2342 23801820 PMC3687325

[24] Dorman D Foster M Fernhoff K Hess P. Canine scent detection of canine cancer: a feasibility study. Vet Med Res Rep. (2017) 8:69–76. 10.2147/VMRR.S148594 30050858 PMC6042482

[25] Hardin DS Anderson W Cattet J. Dogs can be successfully trained to alert to hypoglycemia samples from patients with type 1 diabetes. Diabetes Ther. (2015) 6:509–17. 10.1007/s13300-015-0135-x 26440208 PMC4674474

[26] Reeve C Cummings E McLaughlin E Smith S Gadbois S. An idiographic investigation of diabetic alert dogs' ability to learn from a small sample of breath samples from people with type 1 diabetes. Can J Diabetes. (2020) 44:37–43.e1. 10.1016/j.jcjd.2019.04.020 31477521

[27] Willis CM Church SM Guest CM Cook WA McCarthy N Bransbury AJ. Olfactory detection of human bladder cancer by dogs: proof of principle study. BMJ. (2004) 329:712. 10.1136/bmj.329.7468.712 15388612 PMC518893

[28] Bomers MK van Agtmael MA Luik H Vandenbroucke-Grauls CMJE Smulders YM A. detection dog to identify patients with *Clostridium difficile* infection during a hospital outbreak. J Infect. (2014) 69:456–61. 10.1016/j.jinf.2014.05.017 24973552

[29] Charles MK Wang Y Zurberg T Kinna J Bryce E. Detecting Clostridioides (Clostridium) difficile using canine teams: what does the nose know? Infect Prev Pract. (2019) 1:100005. 10.1016/j.infpip.2019.100005 34368671 PMC8336037

[30] Guirao A Molins L Ramón I Sunyer G Viñolas N Marrades R. Trained dogs can identify malignant solitary pulmonary nodules in exhaled gas. Lung Cancer. (2019) 135:230–3. 10.1016/j.lungcan.2019.06.008 31235316

[31] Guest C Harris R Sfanos KS Shrestha E Partin AW Trock B. Feasibility of integrating canine olfaction with chemical and microbial profiling of urine to detect lethal prostate cancer. PLoS ONE. (2021) 16:e0245530. 10.1371/journal.pone.0245530 33596212 PMC7888653

[32] Horvath G Andersson H Nemes S. Cancer odor in the blood of ovarian cancer patients: a retrospective study of detection by dogs during treatment, 3 and 6 months afterward. BMC Cancer. (2013) 13:396. 10.1186/1471-2407-13-396 23978091 PMC3765942

[33] Taverna G Tidu L Grizzi F Stork B Mandressi A Seveso M. Highly-trained dogs' olfactory system for detecting biochemical recurrence following radical prostatectomy. Clin Chem Lab Med CCLM. (2016) 54:e67–70. 10.1515/cclm-2015-0717 26402886

[34] Vaarno J Myller J Bachour A Koskinen H Bäck L Klockars T. A detection dog for obstructive sleep apnea: could it work in diagnostics? Sleep Breath. (2020) 24:1653–6. 10.1007/s11325-020-02113-1 32468236 PMC7679355

[35] Aviles-Rosa EO Gallegos SF Prada-Tiedemann PA Hall NJ. An automated canine line-up for detection dog research. Front Vet Sci. (2021) 8:775381. 10.3389/fvets.2021.775381 35071382 PMC8771161

[36] Grandjean D Sarkis R Lecoq-Julien C Benard A Roger V Levesque E. Can the detection dog alert on COVID-19 positive persons by sniffing axillary sweat samples? A proof-of-concept study. PLoS ONE. (2020) 15:e0243122. 10.1371/journal.pone.0243122 33301539 PMC7728218

[37] Elliker KR Sommerville BA Broom DM Neal DE Armstrong S Williams HC. Key considerations for the experimental training and evaluation of cancer odour detection dogs: lessons learnt from a double-blind, controlled trial of prostate cancer detection. BMC Urol. (2014) 14:22. 10.1186/1471-2490-14-22 24575737 PMC3945616

[38] Spinhirne JP Koziel JA Chirase NK. Sampling and analysis of volatile organic compounds in bovine breath by solid-phase microextraction and gas chromatography–mass spectrometry. J Chromatogr A. (2004) 1025:63–9. 10.1016/j.chroma.2003.08.062 14753672

[39] Maurer D Koziel J Engelken T Cooper V Funk J. Detection of volatile compounds emitted from nasal secretions and serum: towards non-invasive identification of diseased cattle biomarkers. Separations. (2018) 5:18. 10.3390/separations5010018

[40] Maurer DL Ellis CK Thacker TC Rice S Koziel JA Nol P. Screening of microbial volatile organic compounds for detection of disease in cattle: development of lab-scale method. Sci Rep. (2019) 9:12103. 10.1038/s41598-019-47907-w 31431630 PMC6702204

[41] Belizário JE Faintuch J Malpartida MG. Breath biopsy and discovery of exclusive volatile organic compounds for diagnosis of infectious diseases. Front Cell Infect Microbiol. (2021) 10:564194. 10.3389/fcimb.2020.564194 33520731 PMC7839533

[42] White BJ Renter DG. Bayesian estimation of the performance of using clinical observations and harvest lung lesions for diagnosing bovine respiratory disease in post-weaned beef calves. J Vet Diagn Invest. (2009) 21:446–53. 10.1177/104063870902100405 19564492

[43] Eskandari E Ahmadi Marzaleh M Roudgari H Hamidi Farahani R Nezami-Asl A Laripour R. Sniffer dogs as a screening/diagnostic tool for COVID-19: a proof of concept study. BMC Infect Dis. (2021) 21:243. 10.1186/s12879-021-05939-6 33673823 PMC7934999

[44] Jendrny P Schulz C Twele F Meller S von Köckritz-Blickwede M Osterhaus ADME. Scent dog identification of samples from COVID-19 patients – a pilot study. BMC Infect Dis. (2020) 20:536. 10.1186/s12879-020-05281-3 32703188 PMC7376324

[45] Gazit I Terkel J. Explosives detection by sniffer dogs following strenuous physical activity. Appl Anim Behav Sci. (2003) 81:149–61. 10.1016/S0168-1591(02)00274-5

[46] Bray EE Otto CM Udell MAR Hall NJ Johnston AM MacLean EL. Enhancing the selection and performance of working dogs. Front Vet Sci. (2021) 8:644431. 10.3389/fvets.2021.644431 34055947 PMC8149746

[47] Tiira K Tikkanen A Vainio O. Inhibitory control – important trait for explosive detection performance in police dogs? Appl Anim Behav Sci. (2020) 224:104942. 10.1016/j.applanim.2020.104942

[48] MacLean EL Hare B. Enhanced selection of assistance and explosive detection dogs using cognitive measures. Front Vet Sci. (2018) 5:236. 10.3389/fvets.2018.00236 30338264 PMC6180148

[49] Concha A Mills DS Feugier A Zulch H Guest C Harris R. Using sniffing behavior to differentiate true negative from false negative responses in trained scent-detection dogs. Chem Senses. (2014) 39:749–54. 10.1093/chemse/bju045 25214467 PMC4201303

